# Prebiotic Therapy with Inulin Associated with Low Protein Diet in Chronic Kidney Disease Patients: Evaluation of Nutritional, Cardiovascular and Psychocognitive Parameters

**DOI:** 10.3390/toxins12060381

**Published:** 2020-06-09

**Authors:** Silvia Lai, Sandro Mazzaferro, Maurizio Muscaritoli, Daniela Mastroluca, Massimo Testorio, Adolfo Perrotta, Ylenia Esposito, Maria Carta, Linda Campagna, Marta Di Grado, Cesarina Ramaccini, Sabrina De Leo, Alessandro Galani, Maria Ida Amabile, Alessio Molfino

**Affiliations:** 1 Department of Translational and Precision Medicine, Sapienza University of Rome, 00185 Rome, Italy; sandro.mazzaferro@uniroma1.it (S.M.); maurizio.muscaritoli@uniroma1.it (M.M.); daniela.mastroluca@uniroma1.it (D.M.); adolfo.perrotta@gmail.com (A.P.); ylenia.esposito87@gmail.com (Y.E.); maria.carta@uniroma1.it (M.C.); linda.cam@libero.it (L.C.); martadigrado@hotmail.com (M.D.G.); cesarina.ramaccini@uniroma1.it (C.R.); S.DeLeo@policlinicoumberto1.it (S.D.L.); mariaida.amabile@uniroma1.it (M.I.A.); alessio.molfino@uniroma1.it (A.M.); 2 Department of Obstetrical-Gynecological Sciences and Urologic Sciences, Unit of Nephrology, Sapienza University of Rome, 00161 Rome, Italy; massimo.testorio@uniroma1.it; 3 Department of Clinical and Experimental Sciences, University of Brescia, 25123 Brescia, Italy; xelainalag@yahoo.it

**Keywords:** chronic kidney disease, low protein diet, inulin, microbiota, metabolic profile, cardiovascular risk, psychocognitive evaluation

## Abstract

A relationship between dysbiotic gut microbiome and chronic kidney disease (CKD) has been recently documented; it contributes to CKD-related complications, including cardiovascular disease. Aim: We tested how a low-protein diet (LPD)—with or without oral inulin supplementation as a prebiotic—modulates some inflammatory, atherosclerosis and endothelial dysfunction indices and nutritional markers, as well as psychocognitive functions in CKD patients. We conducted a prospective, case–control study on CKD patients on conservative therapy, divided in two groups: the intervention group treated with LPD (0.6 g/kg/day) plus inulin (19 g/day) and a control group treated with LPD without inulin, for six consecutive months. Clinical and hematochemical parameters as well as instrumental, and psychocognitive assessments (by SF-36 survey and MMSE, HAM-D, BDI-II) were recorded in all the participants at baseline (T0), at three months (T1) and at six months (T2). A total of 41 patients were enrolled: 18 in the intervention group and 23 in the control group. At T2, in both groups, we observed a significant reduction of serum nitrogen and phosphorus (*p* ≤ 0.01) and serum uric acid (*p* ≤ 0.03), and an improvement in metabolic acidosis (bicarbonates, *p* ≤ 0.01; base excess, *p* ≤ 0.02). Moreover, at T2 the intervention group showed a reduction in serum insulin (*p* = 0.008) and fasting glucose levels (*p* = 0.022), HOMA-IR (*p* = 0.004), as well as lower total serum cholesterol (*p* = 0.012), triglycerides (*p* = 0.016), C-reactive protein (*p* = 0.044) and homocysteine (*p* = 0.044) and higher HDL (*p* < 0.001) with respect to baseline. We also observed a significant amelioration of some quality of life and functional status indices (SF-36 survey) among the intervention group compared to controls, without a significant improvement in the cognitive state (MMSE). On the other hand, an amelioration in mood (by HAM-D and BDI-II) was found in the intervention group and in controls (only by BID-II). In conclusion, LPD in association with oral inulin supplementation improved glycemic and lipid metabolism and ameliorated the systemic inflammatory state, likely reducing cardiovascular risk in CKD patients and this may represent a promising therapeutic option, also improving quality of life and mood.

## 1. Introduction

The gut microbiota contributes to host metabolism, nutritional status and immunity, and carries out a protective action against different type of infections [1], being represented by saccharolytic bacteria, that are predominant in healthy conditions and produce most of short chain fatty acids (SCFA), whose beneficial effects consist in regulating glycemic and lipid metabolism, maintaining intact the intestinal barrier and the intestinal pH, and regulating immune system and inflammatory response [2,3]. Several acute and chronic diseases—including chronic kidney disease (CKD), as we recently observed—may determine dysbiosis [3], increasing inflammation and oxidative stress, favoring CKD-toxicity and disease progression as well as increasing cardiovascular risk in this population [4]. Although the main cause of intestinal dysbiosis in CKD patients remains unknown, several hypotheses have been formulated, i.e. the increased transit of urea transformed into ammonia and ammonium hydroxide, the increased intestinal pH and altered intestinal barrier with bacterial translocation and subsequent endotoxemia, determining inflammation [4,5]. Malnutrition, edema, fluid overload and intestinal wall congestion alter the intestinal blood flow and the colonic fecal transit, increasing intestinal barrier permeability [6]. Low fiber diet, the main energy substrate for intestinal bacteria that produce SCFA, favors the increase of proteolytic bacteria [7]. Frequent use of antibiotics and metabolic acidosis increase the catabolism of muscle proteins by promoting insulin resistance with an increase in microbiota species with proteolytic metabolism at the expense of saccharolytic metabolism [7,8]. Recently, new therapeutic approaches based on the modulation of the microbiota have been sought, consisting in the administration of specific diet in addition to prebiotics and/or probiotics [3,9,10]. Microbiota modulation may represent a novel therapeutic strategy, that may be achieved by nutritional intervention with low protein diet (LPD), which was able to lower uremic toxins produced by gut microbiota in non-dialysis CKD patients [2], and by oral administration of prebiotics, such as inulin [3,11]. Inulin has a neutral taste, little side effects and has shown to increase the growth of saccharolytic bacteria [1,12,13]. In particular, we previously demonstrated in CKD patients that LPD in association with inulin was able to modulate gut microbiota and ameliorate some metabolic parameters [3], although no conclusive data are available on the effects on cardiovascular and nutritional indices.

AIM: To evaluate the effects overtime of the LPD with or without oral inulin supplementation, as prebiotic, on inflammatory, atherosclerosis and endothelial dysfunction indices, on nutritional biomarkers, as well as on psychocognitive functions in CKD patients.

## 2. Results

Patient characteristics at baseline (T0) are shown in Table 1. A total of 41 CKD patients (25 males) with a mean age of 61.38 ± 12.36 years were enrolled, 18 patients were treated with LPD (0.6 g/kg/day) plus inulin (19 g/day) (LPD + Inulin Group) and 23 patients, serving as controls, were treated only with LPD (0.6 g/kg/day) (LPD Group). Both groups continued the treatment for 6 consecutive months. At T0, no differences were observed between the two groups in terms of patient’s characteristics including demographic, anthropometric and metabolic parameters (Table 1) and regarding therapies that were all continued during the study period.

### 2.1. Changes Observed between T0 and T1 in LPD Group

At T1 (after 3 months), we observed a significant increase in serum bicarbonate (*p* = 0.013) and base excess (BE) (*p* = 0.045), and a reduction of serum uric acid (SUA) (*p* = 0.049) and serum phosphorus (*p* = 0.047) with a reduction of serum nitrogen (*p* = 0.055) (Table 2), whereas no significant changes in C-reactive protein (CRP) levels were detected.

### 2.2. Changes Observed between T0 and T1 in LPD + Inulin Group

At T1, patients treated with LPD plus inulin showed a reduction of SUA (*p* = 0.046), serum sodium (*p* = 0.024), serum phosphorus (*p* = 0.016) and serum nitrogen (*p* = 0.002), in addition to a significant increase of serum bicarbonates and BE (*p* = 0.007 and *p* = 0.012, respectively) (Table 3). No significant changes in CRP levels were observed.

### 2.3. Changes Observed between T0 and T2 in LPD Group

At T2 (after 6 months), patients treated with LPD showed compared to baseline a significant reduction of SUA (*p* = 0.009), serum nitrogen (*p* = 0.010), serum phosphorus (*p* = 0.008) and a significant increase of serum bicarbonate (*p* = 0.006) and BE (*p* = 0.021). In addition. waist circumference (WC) and body mass index (BMI) tended to reduce (*p* = 0.048, *p* = 0.055, respectively) (Table 2). No significant changes were observed within this group in lipid and glucose/insulin profile, as well as in inflammation and in homocysteine levels.

### 2.4. Changes Observed between T0 and T2 in LPD + Inulin Group

At T2, patients treated with LPD plus inulin have shown compared to baseline a significant reduction of SUA (*p* = 0.033), serum nitrogen (*p* = 0.003), serum phosphorus (*p* = 0.005) and a significant higher concentration of serum bicarbonate (*p* = 0.010) and BE (*p* = 0.009) (Table 3); moreover, we observed an amelioration of lipid and glucose/insulin profile with a significant reduction in the following parameters: serum insulin (*p* = 0.008) and glucose (*p* = 0.022), homeostasis model assessment-insulin resistance (HOMA-IR) (*p* = 0.004), total cholesterol (*p* = 0.012) and triglycerides (*p* = 0.016), whereas an increase of HDL cholesterol (*p* < 0.001) (Table 3). We also found a significant reduction of serum CRP (*p* = 0.044) and homocysteine (*p* = 0.044) (Table 3). As for LPD Group, WC and BMI tended to reduce (*p* = 0.049 and p = 0.061, respectively) (Table 3).

### 2.5. Differences in Metabolic and Clinical Parameters between LPD Group and LPD + Inulin Group at T2

At T2, we observed in LPD + Inulin Group significant lower levels of serum insulin (*p* = 0.015), total cholesterol (*p* < 0.001), triglycerides (*p* = 0.044), whereas higher HDL cholesterol (*p* = 0.020) when compared to LPD Group (Table 4). Furthermore, serum homocysteine, as well as renal resistive index (RRI) tended to be lower (*p* = 0.060, *p* = 0.056, respectively) in LPD + Inulin Group when compared to LPD Group (Table 4). No difference was observed among the other parameters studied at baseline.

### 2.6. Changes in Psychocognitive Parameters between T0 and T2 in the Two Groups

In the LPD + Inulin Group, a significant amelioration was observed between T0 and T2 in the following short form (SF)-36 health survey items: physical functioning (*p* = 0.028), bodily pain (*p* = 0.034), social functioning (*p* = 0.007), general health perception (*p* < 0.001). Moreover, no differences in mini-mental state examination (MMSE) scores were detected (*p* = 0.086), whereas a reduction in Hamilton depression rating scale (HAM-D) (*p* < 0.001) and Beck depression inventory (BDI)-II score (*p* = 0.028) was present (Table 5).

In the LPD Group, non significant changes were documented between T0 and T2 in SF-36 health survey items, MMSE and HAM-D score, whereas a reduction of BDI-II score (*p* = 0.025) was present.

At T2, no difference in terms of psychocognitive parameters was seen between the two groups.

## 3. Discussion

Nutritional therapy represents an important strategy in the multidisciplinary approach to CKD patients and it should be aimed at reducing the daily intake of protein, phosphorus and sodium, providing an adequate nutrients intake and avoiding the development of protein–energy wasting [14]. The role of the LPD in slowing the progressive loss of renal function has been much debated [15], and currently numerous studies showed that LPD was able to reduce the progression of CKD and proteinuria without inducing protein–energy wasting or cachexia [16,17,18].

In our study, we showed a reduction of WC without a significant reduction of BMI and a significant improvement of metabolic acidosis, which in turn could decrease muscle protein catabolism, insulin resistance, mineral metabolism disorders and inflammation [14]. Moreover, the metabolic acidosis may increase cardiovascular risk and favor CKD progression, determining an increase in aldosterone concentration, endothelin and angiotensin II associated with vascular and renal fibrosis [19]. In addition, metabolic acidosis may increase the production of ammonium ions in the kidney with activation of the inflammatory and complement cascade and consequent renal damage [19]. Therefore, LPD decreases the endogenous production of non-volatile acids, allowing to counteract the tendency to metabolic acidosis in patients with CKD. We also showed a significant reduction of serum nitrogen in both groups, with a clinically relevant variance observed overtime. In parallel, we documented a significant reduction in serum phosphorus, known as independent and reversible cardiovascular risk factor associated with increased cardiovascular events and mortality [20]. In fact, hyperphosphoremia is involved in the pathogenesis of vascular calcifications and endothelial dysfunction, both known as cardiovascular risk factors in CKD population [21]. In patients treated with LPD plus inulin, we found an improvement of some cardiovascular “traditional” and “non-traditional” risk factors, including metabolic, nutritional and inflammatory indices. In fact, we showed an improvement in the lipid and glucose profile with a reduction of HOMA-IR, serum insulin and glucose, in addition to a reduction in total cholesterol and triglycerides and an increase in HDL cholesterol, all these known as cardiovascular protective factors [22]. The intestinal dysbiosis associated with CKD involves the proliferation of proteolytic bacteria with loss of intestinal barrier integrity [3]. The prebiotic therapy, by modulating the growth and activity of the intestinal microbiota, can favor saccharolytic bacteria with the production of SCFA exerting different effects both at intestinal and systemic level [2]. In the intestine, prebiotics stimulate the proliferation of symbiotic bacteria, Bifidobacteria, and may restore the integrity of the intestinal barrier [23]. At the systemic level, prebiotics act on different receptors and modulate the transcription of genes involved in lipid and carbohydrate metabolism [23]. In particular, the systemic effectiveness of prebiotics is due to the wide distribution of a class of G protein-coupled receptors on which SCFAs are bound, called FFAR2 and FFAR3 (free fatty acid receptor 2 and 3) expressed in the intestine, in skeletal muscle, in adipose tissue, in the liver and on the surface of monocytes and macrophages [23]. In the colon, SCFAs stimulate the release of various hormones such as glucagon like peptide 1 and peptide YY (PYY), which work by normalizing post prandial plasma glucose levels by stimulating insulin secretion. Moreover, the intestinal PYY hormone induces the sense of satiety and stimulates the uptake of glucose by skeletal muscle. Several studies have shown that prebiotics are able to regulate blood insulin levels and lipogenesis [20,21]. Elevated levels of glucose and insulin enhance the transcription of genes involved in lipogenesis and determine an increase in circulating lipids, highlighting that the normalization of the lipid profile may also derive from a reduction in serum insulin [22]. The SCFA and indirectly also the prebiotics are able to act on the liver by inhibiting lipogenesis and stimulating the oxidation of fatty acids. This phenomenon is associated with the reduction of cholesterol and triglyceride levels [1]. A similar effect is obtained by the reduction of intestinal pH by organic acids, which makes bile acids less soluble and less absorbable by the intestine; this determines a greater hepatic synthesis of bile acids and a greater hepatic uptake of cholesterol, with a consequent decrease in serum levels [20].

In our study, in both groups, we showed a reduction of WC which is generally associated with improvement in metabolism. The “gut microbiota–brain axis” is a bidirectional signaling axis able to influence body weight through a modulation of energy expenditure, appetite and storage [24,25,26]. In particular, excess abdominal fat is strictly related to higher cardio-metabolic risk [27]. Waist circumference is generally utilized as a surrogate of visceral fat, whose increased values are associated with higher cardiometabolic risk [27]. Interestingly, we found a significant reduction of CRP levels only in patients who received LPD plus inulin. Inflammation is known to promote the development of endothelial dysfunction and accelerated atherosclerosis by altering the function and structure of vascular smooth muscle cells [28,29]. In fact, the reduction of the inflammatory state was also accompanied by a reduction of the markers of endothelial dysfunction in patients treated with LPD plus inulin, as SUA and RRI, with a significant reduction of serum homocysteine, an additional well-known risk factor related to cardiovascular and cerebrovascular events [30]. We also showed a significant improvement in some quality of life domains in patients treated with LPD plus inulin, with an amelioration in mood, confirmed also in patients treated with LPD only. Evaluation of psychological status is an important aspect in the treatment of CKD patients. In fact, patients with CKD may present depressive symptoms, probably related to the awareness to develop end stage renal disease. Depression is associated with a reduced quality of life, sexual dysfunction, lack of adherence to medical and dietary therapy [31]. Moreover, especially in older adults, cognitive deficits may also be characterized by executive and motor slowing and by memory and language deficits [32]. All these conditions importantly reduce the quality of life and may negatively impact on patient compliance and clinical outcomes. For this reason, among CKD patients a psychological evaluation, by administering appropriate diagnostic tests, appears complementary to the nephrology and nutritional assessment, allowing physicians to evaluate the patients’ needs in a more comprehensive way.

Our study has limitations, including the relatively limited number of patients studied, although they all concluded the 6-month study period. In addition, some patients were, since the beginning of the study, on treatments with potential impact on different metabolic indices that may have possibly confounded the results. We did not assess body composition to evaluate changes in adiposity and muscle mass overtime.

## 4. Conclusions

In conclusion, the results obtained in the present study suggest that inulin supplementation in association with LPD improved lipid and glucose metabolism and reduced systemic inflammation, thus representing a possible therapeutic strategy aimed at reducing some “traditional” and “non-traditional” risk factors involved in cardiovascular risk of CKD patients. This intervention was also associated with improvement in psychocognitive parameters that should be considered in the management of CKD patients to improve quality of life and well-being.

## 5. Materials and Methods

The study protocol was approved (19 April 2017) by the Local Clinical Research Ethics Committee (Sapienza University—Azienda Policlinico Umberto I, Rome, Italy) with protocol number 302/17. The study conforms to the principles outlined in the Declaration of Helsinki and we obtained a written consent by each patient enrolled.

### 5.1. Study Design and Subjects

We conducted an interventional prospective controlled study on CKD patients at the University Hospital Azienda Policlinico Umberto I, Sapienza University of Rome, Italy. Patients were divided in two groups: the intervention group was treated for 6 consecutive months with LPD (0.6 g/kg/day) plus oral inulin supplementation (19 g/day) (LPD + Inulin Group) and the control group was treated only with LPD (0.6 g/kg/day) (LPD Group) for the same duration. The two groups were matched for gender and estimated glomerular filtration rate (eGFR). Patients were enrolled from October 2018 to January 2019. The LPD was developed by an expert dietician who prescribed a personalized dietetic plan for each patient. The LPD was characterized by an intake of proteins with high biologic value, including protein-free food products, with a low consumption levels of sodium, potassium, phosphorus and low acid-inducing dietary proteins, providing 30–35 Kcal/Kg/day [3]. Adherence to the diet was assessed every three months evaluating urinary nitrogen. Clinical, hematochemical and instrumental measurements were performed in all patients, before starting the LPD with or without inulin (baseline-T0), at 3 months (T1) and at 6 months (T2). As for routine clinical assessment, all the patients also performed cardiovascular evaluation by transthoracic echocardiography, blood pressure and ankle/brachial index measurements [33,34].

### 5.2. Patients

We considered patients with CKD stage 3–4G kidney disease improving global outcomes (KDIGO) (15 mL/min ≤ eGFR ≤ 60 mL/min) on conservative therapy, chronically treated with lipid lowering, anti-hypertensive and anti-platelet therapies, as well as supplemented with calcium, calcitriol and phosphate binders. The eGFR was calculated with the abbreviated Chronic kidney disease-epidemiology formula (CKD-EPI) [35].

We excluded patients aged <18 or >80 years, patients with severe heart failure (NYHA class IV) or acute heart failure and congenital heart disease, severe ongoing infections, cancer, liver and intestinal failure, and those with missing data and without written consent.

#### 5.2.1. Laboratory Measurements

We measured in all patients, after 12 h of fasting, the levels of serum creatinine (mg/dL), serum nitrogen (mg/dL), SUA (mg/dL), serum glucose (mg/dL), insulin (µU/mL), serum electrolytes, total serum cholesterol (mg/dL), triglycerides (mg/dL), HDL (mg/dL), CRP (μg/L), homocysteine (µmol/L) using standard automated techniques. HOMA-IR was calculated as indicated by Mathew et al. [36]. Arterial blood gas was performed using a blood gas analyzer (Nova Phox Plus C, Prospect Street, Waltham, MA, USA).

#### 5.2.2. Anthropometric Assessments

Body weight was determined to the nearest 0.1 kg using a calibrated digital scale. BMI was calculated from a person’s weight and height (weight (kg)/[height (m)]^2^). We measured the WC by placing a tape measure horizontally around the abdomen at the level of the iliac crest at the end of a normal expiration.

#### 5.2.3. Carotid Intima-Media Thickness Assessment (IMT) and Flow-Mediated Dilation Brachial Artery (FMD)

All patients were studied with the high-resolution B-mode ultrasound machine Toshiba Aplio xV (Toshiba Aplio xV, Toshiba American Medical Systems, Inc., Tustin, CA, USA) equipped with a 5- to 12-MHz linear transducer, following a standardized protocol [37], to measure IMT at 3 points on the far walls of both left and right distal common carotid arteries. The mean IMT was calculated as the average IMT on both sides and it was considered normal an IMT value between 0.55 and 0.9. FMD was assessed as described by Celermajer [38] as: (diameter post-hyperemia-basal diameter/basal diameter) × 100. A value of FMD greater than 10% was considered normal.

#### 5.2.4. Renal Resistive Index (RRI)

Renal resistive index was assessed by the same ultrasound machine equipped with a 3–3.5 MHz convex transduce, as previously described [3]. In summary, RRI was calculated as: [1-(end-diastolic velocity ÷ maximal systolic velocity)] × 100 [20]. The intra-reader correlation coefficient for RRI was 0.97, whereas the inter-reader was 0.92.

#### 5.2.5. Psychological and Cognitive Tests

All patients performed the following standardized tests.

##### Short Form (SF-36) Health Survey

The SF-36 health survey is a 36-item patient-reported survey and represents a measure of health status, including the following sections: Physical functioning, vitality, general health perceptions, bodily pain, physical role functioning, social role functioning, emotional role functioning and mental health [39].

##### The Mini-Mental State Examination (MMSE)

The MMSE investigates cognitive and intellectual difficulties and is often used as a screening tool in patients with different neuropsychological syndromes. It consists in 30 items, referring to seven different cognitive areas: orientation in space, orientation in time, attention and calculation, recording of words, language, commemoration and constructional praxis [40].

##### The Hamilton Depression Rating Scale (HAM-D)

The HAM-D is used to determine the level of depression of patients [41]. HAM-D is composed of 21 items, but the scoring is based on the first 17. Eight items are scored on a 5-point scale, ranging from 0 = not present to 4 = severe. Nine are scored from 0–2.

##### Beck Depression Inventory-II (BDI-II)

The BDI-II is a self-assessment questionnaire used to assess the level of depression investigating symptoms such as hopelessness and irritability, guilt or feelings of being punished, fatigue, weight loss, pain and lack of interest in sex [42].

### 5.3. Statistical Analyses

All continuous variables were expressed as mean ± standard deviation, categorical variables were expressed as number (percentage). The normality of variables was tested using the Shapiro–Wilk method. Group comparisons were performed by Student’s unpaired 2-tailed *t*-test or by Mann–Whitney U test, as appropriate. A p value < 0.05 was considered statistically significant.

Data management and analysis were performed using IBM^®^ SPSS^®^ Statistics 22.0 for Windows^®^ software (IBM Corporation, New Orchard Road Armonk, New York, NY, United States).

## Figures and Tables

**Table 1:** Patient characteristics at T0 (baseline). Data are show as mean ± standard deviation or number (%). Abbreviations: LPD—low protein diet; BMI—body mass index; WC—waist circumference; eGFR—estimated glomerular filtration rate; HOMA-IR—homeostasis model assessment: insulin resistance; CRP—C-reactive protein; BE—base excess; HCO_3_^−^—serum bicarbonate; ABI—ankle brachial index; IMT—intima media thickness; FMD—flow mediated dilation; RRI—renal resistive index; BDI-II—Beck depression inventory-II; HAM-D—Hamilton depression rating scale; MMSE—mini-mental state examination.

Parameter	LPD + Inulin Group N = 18	LPD Group N = 23	*p* Value
Male	10 (54%)	15 (57%)	
BMI (kg/m^2^)	29.01 ± 3.95	28.90 ± 3.46	0.07
WC (cm)	105.0 ± 10.6	104.0 ± 9.7	0.756
Age (years)	62.88 ± 7.37	60.0 ± 9.9	0.264
Serum creatinine (mg/dL)	2.64 ± 0.72	2.27 ± 0.42	0.177
eGFR (mL/min)	24.72 ± 6.92	29.61 ± 8.28	0.786
Serum nitrogen (mg/dL)	112.76 ± 29.71	113.00 ± 37.14	0.982
Serum uric acid (mg/dL)	6.15 ± 1.01	5.92 ± 1.32	0.544
Serum glucose (mg/dL)	97.94 ± 10.07	95.15 ± 11.28	0.871
Serum phosphorus (mg/dL)	4.62 ± 0.44	4.89 ± 0.65	0.139
Serum sodium (mEq/L)	143.45 ± 4.7	142.10 ± 1.97	0.219
Total cholesterol (mg/dL)	201.16 ± 45.03	211.55 ± 37.12	0.220
HDL cholesterol (mg/dL)	43.86 ± 7.11	46.05 ± 8.42	0.376
Serum triglycerides (mg/dL)	130.11 ± 49.80	120.11 ± 38.80	0.473
Serum insulin (µU/mL)	12.34 ± 4.53	9.63 ± 5.42	0.381
HOMA–IR	2.95 ± 1.21	2.26 ± 1.33	0.360
CRP (mg/L)	5.68 ± 3.63	5.23 ± 2.98	0.665
BE (mEq/L)	−3.38 ± 3.68	−3.06 ± 3.28	0.307
HCO3^−^ (mEq/L)	22.41± 3.39	21.53 ± 3.38	0.381
Serum homocysteine (mg/dL)	23.87 ± 12.31	24.95 ± 14.11	0.798
IMT (mm)	0.95 ± 0.22	0.92 ± 0.16	0.987
FMD (%)	9.73 ± 6.32	13.99 ± 7.61	0.944
RRI	0.71 ± 0.05	0.69 ± 0.09	0.403
BDI-II	7.18 ± 5.84	8.05 ± 5.90	0.941
HAM-D	13.62 ± 5.11	11.72 ± 5.72	0.762
MMSE	25.58 ± 2.76	26.11 ± 2.39	0.235

**Table 2:** Metabolic and clinical parameters at T0 (baseline), T1 (after 3 months of LPD), and T2 (after 6 months of LPD). Data are show as mean ± standard deviation. Abbreviations: BE—base excess; HCO_3_^−^—serum bicarbonate; WC—waist circumference; BMI—body mass index. * T0 vs T1; ^#^ T0 vs T2.

Parameter	T0	T1	T2	*p* Value *	*p* Value ^#^
BE (mmol/L)	−3.06 ± 3.28	−1.24 ± 2.67	−1.02 ± 2.45	0.045	0.021
HCO_3_^−^(mEq/L)	21.53 ± 3.38	23.89 ± 2.81	24.06 ± 2.56	0.013	0.006
Serum uric acid (mg/dL)	5.92 ± 1.32	5.16 ± 1.23	4.98 ± 1.01	0.049	0.009
Serum nitrogen (mg/dL)	113.00 ± 37.14	89.39 ± 43.85	85.06 ± 34.12	0.055	0.010
Serum phosphorus (mg/dL)	4.89 ± 0.65	4.58 ± 0.33	4.4 ± 0.54	0.047	0.008
WC (cm)	104.0 ± 9.7	101.2 ± 10.1	99.1 ± 6.5	0.342	0.048
BMI (kg/m^2^)	28.90 ± 3.46	27.67 ± 3.09	27.57 ± 2.45	0.210	0.055

**Table 3:** Metabolic and clinical parameters at T0 (baseline), T1 (after 3 months of LPD + inulin) and T2 (after 6 months of LPD + inulin). Data are show as mean ± standard deviation. Abbreviations: LPD—low protein diet; BE—base excess; HCO_3_^−^—bicarbonates; CRP—C-reactive protein; HOMA IR—homeostasis model assessment: insulin resistance; HDL—high-density lipoprotein; WC—waist circumference; BMI—body mass index. * T0 vs T1; ^#^ T0 vs T2.

Parameter	T0	T1	T2	*p* Value *	*p* Value ^#^
BE (mmol/L)	−3.38 ± 3.68	−0.78 ± 2.03	−0.68 ± 1.98	0.012	0.009
HCO_3_^−^ (mEq/L)	22.41± 3.39	25.04 ± 2.01	25.36 ± 3.16	0.007	0.010
Serum uric acid (mg/dL)	6.15 ± 1.01	5.41 ± 1.13	5.33 ± 1.2	0.046	0.033
Serum nitrogen (mg/dL)	112.76 ± 29.71	85.26 ± 19.88	83.23 ± 26.89	0.002	0.003
Serum phosphorus (mg/dL)	4.62 ± 0.44	4.2 ± 0.55	4.12 ± 0.57	0.016	0.005
Serum sodium (mmol/L)	143.45 ± 4.7	140.23 ± 3.37	139.02 ± 3.56	0.024	0.024
CRP (mg/L)	5.68 ± 3.63	3.84 ± 2.37	3.67 ± 1.88	0.080	0.044
Serum homocysteine (mg/dL)	23.87 ± 12.31	20.33 ± 9.98	16.34 ± 9.11	0.350	0.044
Serum insulin (µU/mL)	12.34 ± 4.53	10.30 ± 5.80	8.48 ± 3.72	0.247	0.008
Serum glucose (mg/dL)	97.94 ± 10.39	91.43 12.30	88.94 ± 12.15	0.095	0.022
HOMA-IR	2.95 ± 1.21	2.72 ± 1.38	1.95 ± 0.68	0.598	0.004
Total cholesterol (mg/dL)	201.16 ± 45.03	185.07 ± 28.67	166.22 ± 33.29	0.209	0.012
HDL cholesterol (mg/dL)	43.86 ± 7.11	46.10 ± 10.69	53.00 ± 8.34	0.464	<0.001
Serum triglycerides (mg/dL)	130.11 ± 49.80	125.0 ± 48.83	97.41 ± 29.21	0.757	0.016
WC (cm)	105.00 ± 10.6	101.83 ± 9.58	98.83 ± 8.85	0.353	0.049
BMI (kg/m^2^)	29.01 ± 3.95	27.80 ± 3.66	27.16 ± 2.12	0.347	0.061

**Table 4:** Metabolic and clinical parameters at T2 (after 6 months of treatment) in the two groups. Data are show as mean ± standard deviation or number (%). Abbreviations: HDL—high-density lipoprotein; RRI—renal resistive index.

Parameter	LPD + Inulin Group N = 18	LPD Group N = 23	*p* Value
Total cholesterol (mg/dL)	166.22 ± 33.29	217.05 ± 31.9	<0.001
HDL cholesterol (mg/dL)	53.00 ± 8.34	46.11 ± 9.62	0.020
Triglycerides (mg/dL)	97.41 ± 29.21	125.00 ± 50.10	0.044
Serum insulin (µU/mL)	8.48 ± 3.72	12.03 ± 4.91	0.015
Serum homocysteine (mg/dL)	16.34 ± 9.11	23.07 ± 12.04	0.056
RRI	0.69 ± 0.04	0.73 ± 0.08	0.060

**Table 5:** Psychocognitive parameters at T0 and T2 in the two groups. Data are show as mean ± standard deviation. Abbreviations: BDI-II—Beck depression inventory-II; HAM-D—Hamilton depression rating scale; MMSE—mini-mental state examination; SF36—short form-36 health survey.

Parameter	LPD + Inulin Group N = 18			LPD Group N = 23		
	T0	T2	*p* Value	T0	T2	*p* Value
BDI-II	7.18 ± 5.84	3.80 ± 3.30	0.028	8.05 ± 5.90	4.29 ± 5.04	0.025
HAM-D	13.62 ± 5.11	7.26 ± 5.93	<0.001	11.72 ± 5.72	9.16 ± 4.94	0.111
MMSE	25.58 ± 2.76	27.15 ± 2.58	0.086	26.11 ± 2.39	25.88 ± 1.40	0.692
SF36 physical functioning	50.52 ± 27.82	72.16 ± 28.68	0.028	63.95 ± 27.14	64.98± 26.92	0.909
SF36 bodily pain	61.86 ± 18.76	76.34 ± 20.43	0.034	64.88 ± 21.60	64.44 ± 21.52	0.945
SF36 social functioning	75.16 ± 16.59	89.98 ± 14.51	0.007	76.88 ± 13.23	82.33 ± 13.42	0.172
SF36 general health perception	44.33 ± 17.28	64.33 ± 16.31	<0.001	45.88 ± 17.34	54.27± 17.20	0.106
